# Thrombomodulin favors leukocyte microvesicle fibrinolytic activity, reduces NETosis and prevents septic shock-induced coagulopathy in rats

**DOI:** 10.1186/s13613-017-0340-z

**Published:** 2017-12-08

**Authors:** Julie Helms, Raphaël Clere-Jehl, Elsa Bianchini, Pierrick Le Borgne, Mélanie Burban, Fatiha Zobairi, Jean-Luc Diehl, Lelia Grunebaum, Florence Toti, Ferhat Meziani, Delphine Borgel

**Affiliations:** 1 UMR INSERM 1176-Universite Paris Sud, Hôpital Bicêtre, 78 rue du Général Leclerc, 94270 Le Kremlin-Bicêtre, France; 2 Réanimation Médicale, Hôpital Européen Georges Pompidou, Assistance Publique Hôpitaux de Paris, 20 Rue Leblanc, 75015 Paris, France; 3 Université de Strasbourg (UNISTRA), Faculté de Médecine, Hôpitaux universitaires de Strasbourg, Service de Réanimation, Nouvel Hôpital Civil, Strasbourg, France; 4 INSERM (French National Institute of Health and Medical Research), UMR 1260, Regenerative Nanomedicine (RNM), FMTS, Strasbourg, France; 5 Service d’Accueil des Urgences, Hôpital de Hautepierre, CHU de Strasbourg, 1 Avenue de Molière, 67200 Strasbourg, France; 6 Laboratoire d’hématologie et hémostase, Hôpital de Hautepierre, CHU de Strasbourg, 1 Avenue de Molière, 67200 Strasbourg, France

**Keywords:** DIC, Immunothrombosis, Microvesicles, NETosis, Septic shock

## Abstract

**Background:**

Septic shock-induced disseminated intravascular coagulation is responsible for increased occurrence of multiple organ dysfunction and mortality. Immunothrombosis-induced coagulopathy may contribute to hypercoagulability. We aimed at determining whether recombinant human thrombomodulin (rhTM) could control exaggerated immunothrombosis by studying procoagulant responses, fibrinolysis activity borne by microvesicles (MVs) and NETosis in septic shock.

**Methods:**

In a septic shock model after a cecal ligation and puncture-induced peritonitis (H0), rats were treated with rhTM or a placebo at H18, resuscitated and monitored during 4 h. At H22, blood was sampled to perform coagulation tests, to characterize MVs and to detect neutrophils extracellular traps (NETs). Lungs were stained with hematoxylin–eosin for inflammatory injury assessment.

**Results:**

Coagulopathy was attenuated in rhTM-treated septic rats compared to placebo-treated rats, as attested by a significant decrease in procoagulant annexin A5^+^-MVs and plasma procoagulant activity of phospholipids and by a significant increase in antithrombin levels (84 ± 8 vs. 64 ± 6%, *p* < 0.05), platelet count (582 ± 157 vs. 319 ± 91 × 10^9^/L, *p* < 0.05) and fibrinolysis activity borne by MVs (2.9 ± 0.26 vs. 0.48 ± 0.29 U/mL urokinase, *p* < 0.05). Lung histological injury score showed significantly less leukocyte infiltration. Decreased procoagulant activity and lung injury were concomitant with decreased leukocyte activation as attested by plasma leukocyte-derived MVs and NETosis reduction after rhTM treatment (neutrophil elastase/DNA: 93 ± 33 vs. 227 ± 48 and citrullinated histones H3/DNA: 96 ± 16 vs. 242 ± 180, mOD for 10^9^ neutrophils/L, *p* < 0.05).

**Conclusion:**

Thrombomodulin limits procoagulant responses and NETosis and at least partly restores hemostasis control during immunothrombosis. Neutrophils might thus stand as a promising therapeutic target in septic shock-induced coagulopathy.

**Electronic supplementary material:**

The online version of this article (10.1186/s13613-017-0340-z) contains supplementary material, which is available to authorized users.

## Background

Septic shock is the most severe form of an acute infection, associated with circulatory, cellular and metabolic abnormalities, all leading to organ dysfunction and a high mortality rate [1]. Septic shock is characterized by uncontrolled host response to pathogen, combining both intense systemic inflammation and exaggerated coagulation activation with defective fibrinolysis, ultimately leading to disseminated intravascular coagulation (DIC) [2].

The crosstalk between inflammation pathways and the coagulation system in septic shock has aroused in the past decades. Engelmann and Massberg [3] indeed described the immunothrombosis concept as an innate immune response induced by the activation of coagulation, leading to thrombin generation and microthrombi formation, necessary for pathogen recognition, containment and destruction. At site of pathogen invasion, activated neutrophils thus constitute a first defense line. They are able to release both procoagulant microvesicles (MVs) and neutrophil extracellular traps (NETs). Microvesicles are submicron plasma membrane vesicles shed by activated or apoptotic cells and therefore constitute a signature of cell responses. In blood, circulating MVs are considered as potent cellular effectors promoting vascular cell activation and participating in the hemostatic equilibrium. They indeed constitute a procoagulant surface and accumulate within the thrombus, but endothelial and leukocyte MVs were also shown to support a fibrinolytic activity [4]. MVs released during septic shock are detrimental to the vascular function. They prompt endothelial and leukocyte activation, induce tissue factor expression and promote inflammatory signaling pathways in vessel walls and cardiac tissues [5]. NETs are able to trap pathogens and constitute a surface able to promote blood coagulation. Recently described as a mesh of depacked chromatin, NETs expose citrullinated histones and enzymes like myeloperoxidase and neutrophil elastase [3, 6]. They promote coagulation, through the contact phase activation, by supporting interactions between neutrophils and platelets, and owing to intrinsic procoagulant properties [7–10]. Immunothrombosis limits pathogen spreading by means of NETs and thrombus formation at the initial site of invasion and thus stands as a new beneficial host defense mechanism against pathogens. However, during septic shock, immunothrombosis may also become deleterious, contributing to major tissue insult and excessive coagulation activation [11–13].

In this context, anticoagulation in septic shock patients might prevent or counteract excessive coagulation and immunothrombosis deregulation [14, 15]. Several studies have, however, indicated that treatment with physiological coagulation inhibitors (such as activated protein C) in septic shock might only be of benefit to patients in severe state [16], since thrombosis may take part to the physiological immune defense responses [8, 14]. In Japan, recombinant human thrombomodulin (rhTM) is given since 2008, as a treatment of septic shock-induced DIC. An international randomized controlled phase III trial evaluating the efficacy of rhTM in septic shock-induced DIC is ongoing (NCT01598831). The reasons why rhTM aroused intensivists’ interest are its anticoagulant properties, but also a promising anti-inflammatory activity [17]. Thrombomodulin is a transmembrane multi-domain glycoprotein mostly expressed by endothelial cells and pivotal for the control of hemostasis [18, 19]. In septic shock, the endothelial expression of thrombomodulin is deeply downregulated, leading to a defect in protein C activation. Indeed, thrombin-rhTM complex catalyzes the activation of protein C and the downstream inactivation of cofactors Va and VIIIa, thereby limiting the amplification of the coagulation cascade [17]. Moreover, the lectin-like domain of rhTM has anti-inflammatory properties by inhibiting the complement cascade and leukocyte adhesion to the endothelium, neutralizing lipopolysaccharide (LPS) and degrading the high-mobility group box 1 (HMGB1) pro-inflammatory proteins [20, 21].

The purpose of this study was therefore to determine whether rhTM could control exaggerated immunothrombosis by studying procoagulant responses and fibrinolysis activity borne by MVs and exploring NETosis in a rat model of peritonitis-induced septic shock.

## Methods

This study was performed with the approval of the Strasbourg Regional Committee of Ethics in Animal Experimentation (CREMEAS, AL/04/05/02/12, France).

Endpoints: During the experiments, animals were euthanized if they had signs of suffering not immediately controlled by anesthetic or analgesic injection. Indeed, following the CLP surgical procedure, we monitored the sepsis severity score described by Shrum et al. [22]. This score takes into account several parameters, including appearance, level of consciousness, activity, response to stimulus, eyes, respiration rates and respiration quality, grading each parameter from 1 to 4. The rat was euthanized if a score greater than 21 was reached or if a score > 3 was reached for the rhythm or the respiratory quality. At the end of the experiment, rats were euthanized with intravenous lethal dose of pentobarbital sodium (100 mg/kg pentobarbital sodium, Ceva Santé Animal, France).

### Septic shock model

Male Wistar rats (Janvier Laboratories, Le Genest-Saint-Isle, France), weighing 350 ± 30 g, were randomly allocated to one of the four groups (10 rats/group): sham–NaCl, sham–TM, CLP–NaCl or CLP–TM. The operator was not blinded for sham and CLP surgery, but was blinded for the treatment (TM or placebo) allocated the day after (Additional file 1: Figure S1).

Rats underwent cecal ligation and puncture (CLP) as previously described [23]. First, rats were anesthetized with 1–2% isoflurane (Baxter S.A.S, Maurepas, France) and analgesia was ensured by Vetergesic^®^ (0.1 mg/kg of body weight; Sogeval). Before skin incision, a subcutaneous injection of 0.5% Xylocaïne (AstraZeneca, Rueil-Malmaison, France) was performed. Briefly, after shaving and under aseptic conditions, a 2-cm midline laparotomy was performed to allow exposure of the cecum. The cecum was partially ligated, punctured with a 21-gauge needle and gently squeezed to extrude a small amount of feces from the perforation site. The cecum was then returned into the peritoneal cavity, and the laparotomy was closed. Before housing in their cages with free water and food access, all rats received a subcutaneous injection of 0.9% NaCl (30 ml/kg of body weight). Septic shock occurred within 16–20 h after CLP and was considered established on the basis of clinical criteria (lethargy, piloerection and glassy eyes). Sham rats underwent the same laparotomy and cecal exposure without ligation and puncture, without any further manipulation, and received also a subcutaneous injection of 0.9% NaCl (30 ml/kg of body weight). After the CLP surgery, the rat was placed in a thermostated chamber at 37 °C and observed for 1 h until his complete awakening. The rat was then isolated in a cage, so that he would not be hurt by his congeners. He had free access to water and to both a nutrient gel and standard food, and carded cotton was placed in the cage to allow the animal to keep warm and hide.

### Treatment, resuscitation and monitoring

After 18 h, once septic shock was established, rats were anesthetized with an intraperitoneal injection of pentobarbital sodium (60 mg/kg, i.p.) and analgesia was ensured as described above. Animals were tracheotomized and lungs ventilated throughout the experiment. The ventilator (Harvard Rodent Ventilator 683, Harvard Instruments, South Natick, MA, USA) was set to maintain a PaCO_2_ of 40 mmHg, and oxygen was added to maintain a PaO_2_ of 100 mmHg. A transit-time ultrasound flow probe of 2.0 mm (Transonic Systmens, Ithaca, NY, USA) was attached to the right carotid artery to continuously measure carotid blood flow (CBF). The left femoral artery was also exposed and used to measure mean arterial pressure (MAP) and to collect blood samples. Hydration was ensured by an intravenous infusion of 0.9% sodium chloride (NaCl) at 0.8 ml/h.

After the surgical procedure, animals were allowed to stabilize for 30 min (min). Septic shock was further confirmed with a MAP below 90 mmHg and elevated plasma lactate level (> 2 mmol/l). An intravenous bolus of either recombinant human thrombomodulin (rhTM: 1 mg/kg, Asahi Kasei, Japan) or 0.9% NaCl was performed. The 1 mg/kg dose of rhTM is considered as a “medium” dose for rats [24].

During the 4-h resuscitation, fluid challenge of CLP rats was performed by bolus of 0.9% NaCl (0.5 ml every 15 min when needed) and norepinephrine was infused to target a MAP above 100 mmHg. After a 240-min resuscitation, rats were bled via the arterial catheter on EDTA (7.2 mg/4 ml) and citrate (0.129 M) anticoagulants under sterile condition. The presence of cecum necrosis and intra-abdominal pus was systematically checked. In sham rats, the absence of intra-abdominal infection was checked as well.

#### Hemostasis and hematological analysis

Poor platelet citrated plasma was obtained after a two-step centrifugation (15 min; 2,500 g). The procoagulant activity of phospholipids through assembly of the prothrombinase complex was analyzed using the STA^®^ Procoag-PPL test kit (Stago, Asnières, France) [25]. LIAPHEN antithrombin kit (Hyphen Biomed, Neuville-sur-Oise, France) is a latex immunoassay used for measuring antithrombin (AT) on citrated plasma, according to the manufacturer’s instructions.

Platelet and leukocyte counts were measured using an automated Sysmex XN20 analyzer (Sysmex corporation, Kobe, Japan).

#### Microvesicle quantification

Microvesicles were isolated after successive centrifugations as previously described [22]. Total microvesicles were assessed after capture onto biotinylated annexin-5 insolubilized on covalently coated streptavidin multi-well plates (Roche, Paris, France). Microvesicle samples (100 μl/well) were incubated for 30 min at 37 °C. After three washes, the amount of insolubilized MVs was measured by prothrombinase assay in standardized medium containing human coagulation factors (1.2 μM FII, 33.3 pM FVa, 11.2 pM FXa, 2.2 mM CaCl2). Variations of absorbance of pNAPEP0216, a thrombin chromogenic substrate (1.52 mM final concentration, Cryopep, Montpellier, France), were detected in kinetic mode using a thermostated spectrophotometer set at 405 nm (VersaMax Molecular Devices, Sunnyvale, CA, USA). The absorbance values were converted into nanomolar phosphatidylserine equivalent (PhtdSer eq.) by reference to a calibration curve using synthetic vesicles (33% w/w PhtdSer and 67% w/w PhtdChol). In the prothrombinase assay, PhtdSer exposed by MVs is the rate-limiting factor of the reaction leading to thrombin generation [26].

#### Fibrinolytic microvesicle activity

After three washes, washed MV pellets were re-suspended in 100 µl HEPES (10 mM, NaCl 140 mM, pH 7.40). Multi-well plates (Thermo Fisher Scientific, Illkirch, France) were coated with 150 µl assay buffer made of 50 mM phosphate, 80 mM NaCl, 2 g/L bovine serum albumin (Eurobio, Courtaboeuf, France) and 0.01% Tween. Fibrinolytic MV activity was assessed on 1/1 MV–plasminogen/CBS0065 samples (plasminogen: South Bend, USA; CBS0065: Stago, Asnières-sur-Seine, France). Microvesicle-free plasma (pellet supernatant) was used a negative control, and urokinase (Euromedica, Diegem, Belgium) was used with a range from 0.005 U to 0.5 U. Absorbance variations were measured for 18 h at 405 and 490 nm at 37 °C with a thermostated spectrophotometer (Versamax). Results are expressed in urokinase concentration.

#### Microvesicle characterization

Microvesicle phenotype was assessed using biotinylated monoclonal antibodies instead of annexin-5. Antibodies (1 μg/well) were directed against characteristic antigens borne by the parental cells: anti-CD61 for platelet GPIIIa (β3), anti-CD45 for leukocyte and anti-CD54 for endothelial cells (BioLegend, San Diego, CA, USA). Prothrombinase assay was performed as described above. Absorbance values obtained with irrelevant control antibodies of identical isotypes were subtracted from those obtained with the specific antibody. Levels of endotoxin were assessed in all preparations of MVs with the Limulus Amebocyte Lysate kit QCL-1000 (Limulus Amebocyte Lysate, Endosafe Products & Services, Charles River Laboratories, L’Arbresle Cedex, France) and were below the detection threshold (< 0.1 endotoxin units/mL).

#### Lung infiltration with leukocytes

After killing, lung tissues were fixed by immersion in 10% phosphate-buffered formalin (Sigma-Aldrich, Darmstadt, Germany) for 8 h at room temperature. Following fixation, tissues were washed three times in PBS. After fixation, lung tissues were placed in 10% sucrose until tissues sink, then 20% overnight. Lung tissues were removed from sucrose, excess was blot off, and tissues were placed into molds. Tissues were then transferred to OCT (optimum cutting temperature compound) chamber and surrounded with OCT. The mold was placed in Styrofoam container, which holds dry ice/2-methyl butane slurry, and the block was allowed to freeze until the OCT becomes white and solid. Eight-µm frozen sections were performed using the Cryostat.

Sections were stained with hematoxylin–eosin (HE) (Sigma-Aldrich, Darmstadt, Germany) to assess tissue inflammation. Tissues were microscopically examined for pulmonary injuries. All slides were analyzed using X 40 magnification blinded to treatment group. Two scientists, both of whom were unaware of the group assignments, described the lung histologic changes as previously described [27]. Ten lung parenchyma in each animal were observed at × 10 objective magnification and graded on a scale of 0–4 (0: absent/appears normal; 1: light; 2: moderate; 3: strong; 4: intense) for congestion, edema, inflammation and hemorrhage. Total score was the sum of the four parameters (0–16 points for each slide).

#### Plasma neutrophil extracellular trap and nucleosome detection

Indirect markers of NETosis (citrullinated histones H3/DNA and neutrophil elastase/DNA complexes) were measured in plasma using a direct ELISA protocol. Briefly, 10 µg/mL of primary antibodies directed against citrullinated histones H3 (Abcam, Cambridge, UK) and neutrophil elastase (NE) (Santa Cruz, Heidelberg, Germany) were added per well on 96-well plates (Roche, Paris, France) and incubated overnight at 4 °C. After two washes, the remaining protein-binding sites in the coated wells were blocked by adding PBS-1% BSA and incubating for 2 h. After two washes, 20 µl plasma and 80 µl of anti-DNA peroxidase substrate (Roche, Boulogne-Billancourt, France) were added in each well, and plates were incubated for 2 h at room temperature. Four washes were performed, and 100 μl of the revealing solution was added in each well. After 30-min incubation, the absorbance was measured at 405 nm wavelength using a spectrophotometer device (VersaMax Molecular Devices, Sunnyvale, CA, USA). It was verified that absorbance values were proportional to the amount of a bound peroxidase-labeled antibody. Results are expressed as OD for 10^9^ PNN/L.

Cell-free circulating DNA (nucleosome) was also measured on plasma samples by Cell Death Detection ELISA^PLUS^ kit (Roche, Boulogne-Billancourt, France) according to the manufacturer’s instructions. Results are expressed in arbitrary units (AU for 10^9^ PNN/L).

### Statistical analysis

The inter-group comparison of hemodynamic parameters was performed by ANOVA repeated measures; the pairwise comparison was made using a Tukey–Kramer adjustment p values. For measurement of MVs, hemostasis parameters, NETosis and organ injury score, a nonparametric analysis by Kruskal–Wallis test with Dunn post hoc test was used. All statistics were performed with Statview software (version 5.0, SAS Institute, Cary, NC, USA). All values were presented as mean ± SD for n experiments, with representing the number of rats. A *p* value < 0.05 was considered statistically significant.

## Results

### rhTM treatment attenuated coagulopathy, by decreasing plasma procoagulant activity and increasing fibrinolytic activity borne by MVs

**Fig. 1:**
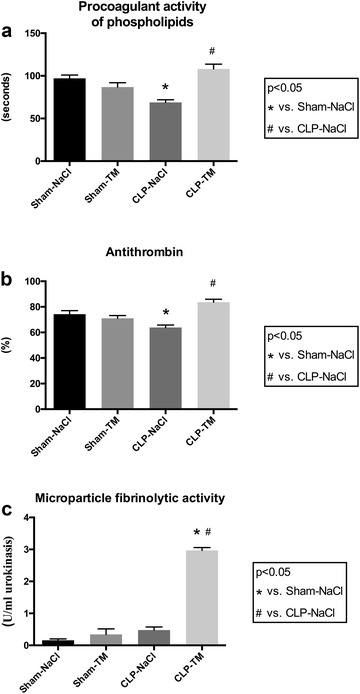
Anticoagulant and fibrinolytic activities. **a** Procoagulant activity of phospholipids was measured in rat plasma using the STA^®^ Procoag-PPL assay. **b** Concentration of antithrombin (AT) was assessed by latex immunoassay in citrated plasma. **c** Fibrinolytic activity borne by total microvesicles was assessed using a microvesicle–plasminogen/CBS0065 samples. *n* = 10 per subset. Results are expressed in mean ± SD. *p < 0.05 versus sham–NaCl group and ^#^ *p* < 0.05 versus CLP–NaCl group

After assessing that rhTM had not significant hemodynamic effect and did not induce bleeding in rats, we have shown that it allowed a significant attenuation of septic shock-induced coagulopathy. Indeed, the concentration of total MVs (Annexin-A5^+^ MVs) was increased by a fivefold range in septic compared to sham rats (29.5 ± 4.2 vs. 6.2 ± 4.4 nM PhtdSer, *p* < 0.05) and was significantly limited by rhTM treatment, values being reduced by 50% (29.5 ± 4.2 vs. 17.3 ± 1.8 nM PhtdSer, *p* < 0.05) (Table 1). Because anionic phospholipids exposed by MVs constitute an additional surface for the assembly of blood coagulation complexes, we determined the procoagulant activity of rat plasma using the procoagulant activity of phospholipids assay (PPL assay), which is inversely related to clotting time. The procoagulant activity was significantly increased in septic shock compared to sham rats, as shown by the shortened time (Fig. 1a). Interestingly, rhTM treatment decreased plasma procoagulant activity in septic rats, while it had no significant effect on sham rats.

**Table 1:** Cell origin of microvesicles emitted by septic and control rats

	Sham–NaCl	Sham–TM	CLP–NaCl	CLP–TM
MVs (nM Eq PhtdSer)				
Annexin-A5^+^ MVs	6.2 ± 4.4	9.1 ± 1.7^b^	29.5 ± 4.2^a^	17.3 ± 1.8^b^
CD45^+^ MVs/leukocytes	0.10 ± 0.08	0.07 ± 0.09^b^	1.8 ± 0.64^a^	0.60 ± 0.51^b^
CD54^+^ MVs	0.69 ± 0.43	1.0 ± 0.36	2.7 ± 1.0^a^	8.4 ± 2.6^a^
CD61^+^ MVs/platelets	0.18 ± 0.10	0.50 ± 0.14	1.0 ± 0.31^a^	0.88 ± 0.18^a^

Septic and control (sham–NaCl and sham–TM) rats were treated by rhTM or 0.9% sodium chloride. Data are expressed as mean ± SD. *p* < 0.05

*CLP* cecal ligation and puncture, *MVs* microvesicles, *PhtdSer* phosphatidylserine, *rhTM* recombinant human thrombomodulin. *n* = 10 for each subset

^a^vs. sham–NaCl

^b^vs. CLP–NaCl

**Fig. 2:**
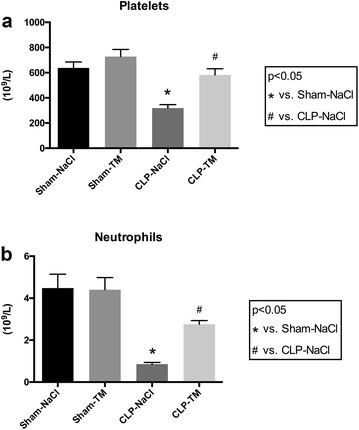
**a** Platelets and **b** neutrophils counts were measured on an automated analyzer. *n* = 10 per subset. Results are expressed in mean ± SD. *p < 0.05 versus sham–NaCl group and ^#^ *p* < 0.05 versus CLP–NaCl group

In order to assess the failure of the anticoagulation pathways in septic shock-induced coagulopathy, we measured plasma antithrombin concentrations in CLP and sham rats. Consistent with the PPL assay, the antithrombin concentration significantly decreased in septic rats compared to sham rats (Fig. 1b). Pharmacological control of coagulopathy by rhTM was also confirmed by the partial restoration of the antithrombin circulating concentration in CLP–TM rats that was significantly higher compared to CLP rats (Fig. 1B). Moreover, compared to sham rats, CLP rats had a significant thrombopenia (and neutropenia), reflecting platelet consumption during septic shock that was significantly improved by rhTM treatment (Fig. 2). It is to be noted that baseline (i.e., before sham or CLP operation) platelet counts were not different in the four groups (data not shown).

Values of MV fibrinolytic activity remained similar in CLP–NaCl and sham–NaCl rats, while a fivefold enhancement in total MVs was measured (see above), suggesting that MVs released during sepsis were mainly procoagulant (Fig. 1C). Most interestingly, rhTM treatment of septic rats resulted in a significant sixfold enhancement in MV fibrinolytic activity (3.0 ± 0.26 vs. 0.48 ± 0.28 U/ml urokinase, *p* < 0.05), thereby indicating that rhTM was able to prompt the generation of a proportion of MVs with restored fibrinolysis activity eventually counteracting septic shock-induced coagulopathy.

### rhTM improved septic shock-induced lung injury

**Fig. 3:**
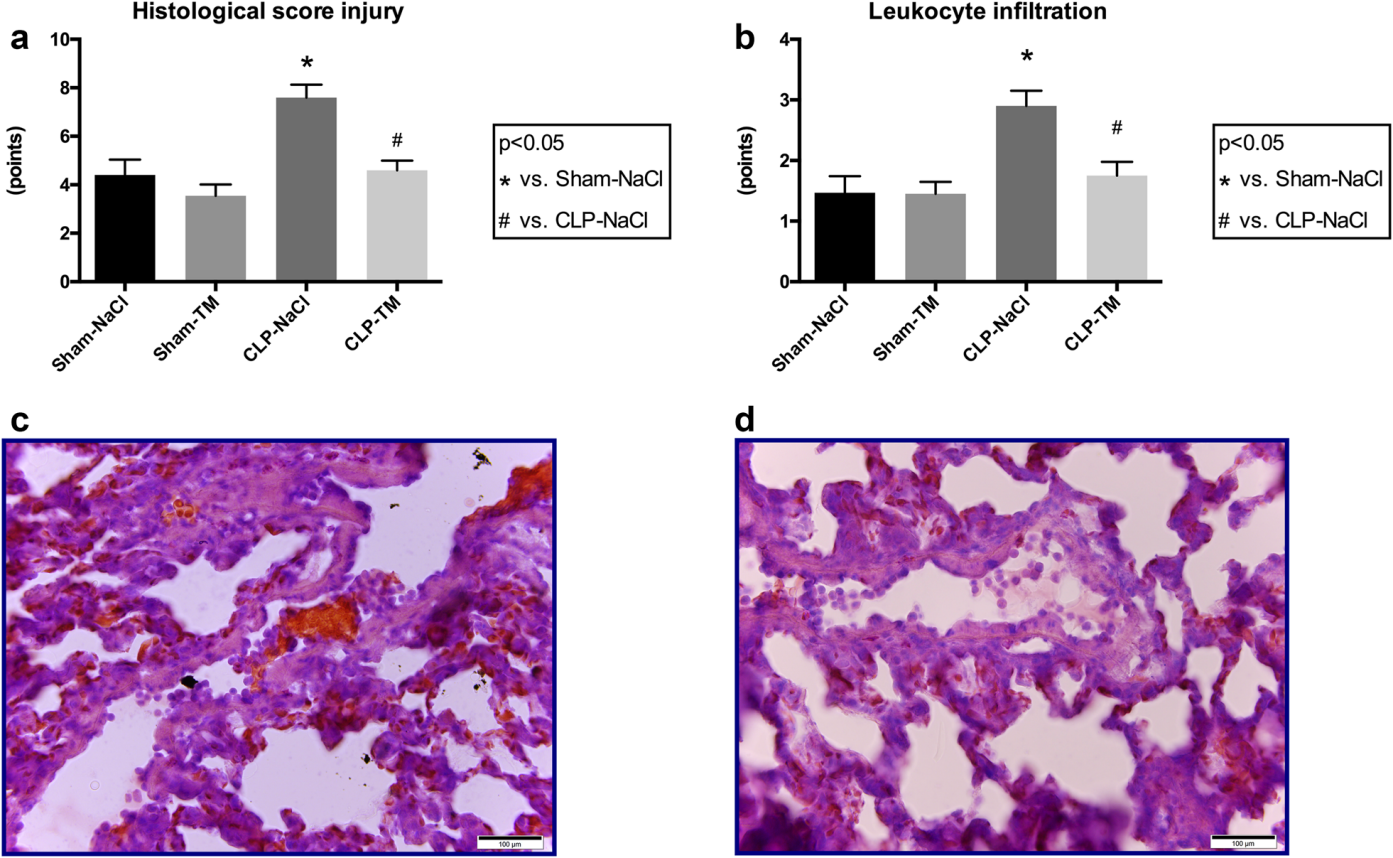
Histological lung injury. Lung sections were stained with hematoxylin–eosin to assess tissue histological injuries. Lung histologic changes were graded on a scale of 0–4 (0: absent/appears normal; 1: light; 2: moderate; 3: strong; 4: intense) for congestion, edema, inflammation and hemorrhage. **a** Total score was the sum of the four parameters (0–16 points for each slide). **b** Leukocyte infiltration is also represented for each group. **c** Lung section representative of CLP–NaCl and (D) CLP–TM groups. Leukocyte infiltration is significantly decreased in lung sections from CLP–TM rats compared to CLP–NaCl rats, leading to a decreased global lung injury score (**a**). *n* = 10 per subset. Results are expressed in mean ± SD. *p < 0.05 versus sham–NaCl group and ^#^ *p* < 0.05 versus CLP–NaCl group

To assess potential cytoprotective and/or anti-inflammatory effects of rhTM on septic shock-induced organ injury, lung sections were analyzed using a reported histological score of lung inflammatory injuries [27]. We showed that congestion, inflammation, edema and hemorrhage were significantly increased in septic rats compared to sham rats, with a significant elevation of the global histological score (8 ± 2 vs. 4 ± 2 points, *p* < 0.05) (Fig. 3a). The global histological score was significantly decreased in septic rats treated by rhTM compared to septic rats treated by placebo (5 ± 2 vs. 8 ± 2 points, *p* < 0.05) (Fig. 3a). Considering individually each determinant of this histological score (congestion, leukocyte infiltration, edema and hemorrhage), only leukocyte infiltration was significantly decreased in septic rats treated by rhTM compared to septic rats treated by placebo (2 ± 1 vs. 3 ± 1 points, *p* < 0.05), whereas the other signs of lung histological injury (congestion, edema and hemorrhage) were not significantly decreased (Fig. 3a–d).

### rhTM treatment decreased the release of septic shock-induced leukocyte-derived microvesicles and systemic NETosis

Using specific antibodies, we identified the cell origin of circulating MVs possibly contributing to septic shock pathophysiology. In CLP–NaCl rats, leukocyte, platelet and endothelial MVs were significantly elevated (*p* < 0.05) compared to sham rats. The rhTM treatment altered the MV pattern, with a significant decrease in the leukocyte MVs-to-leukocyte count ratio (CD45^+^-MVs/leukocytes: 0.60 ± 0.51 vs. 1.8 ± 0.64 nM PhtdSer, *p* < 0.05), while the platelet MVs-to-platelet count ratio (CD61^+^-MVs/platelets) was not significantly different (Table 1).

**Fig. 4:**
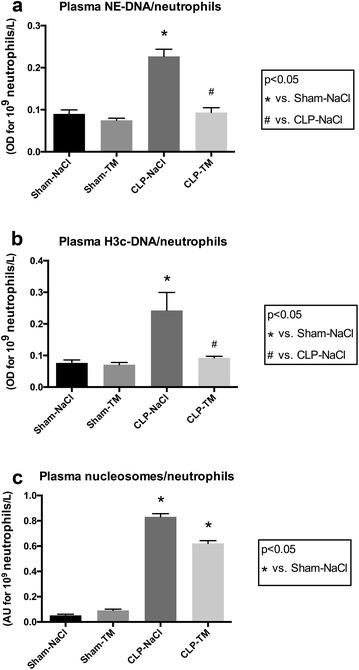
Plasma neutrophil extracellular traps and nucleosomes. NETs were measured in plasma using indirect markers: **a** neutrophil elastase (NE)/DNA complexes and **b** citrullinated histones H3 (H3c)/DNA complexes. **c** Nucleosomes were also measured on plasma samples. Results were reported to neutrophil ratio because of the significant neutropenia observed in septic groups. *n* = 10 per subset. Results are expressed in mean ± SD. *p < 0.05 versus sham–NaCl group and ^#^ *p* < 0.05 versus CLP–NaCl group

Consistent with major sepsis inflammation, systemic NETosis was detected in the plasma of CLP–NaCl rats. Concentrations of plasma nucleosomes, citrullinated histone H3/DNA complexes and neutrophil elastase/DNA complexes were significantly increased in septic compared to sham rats (nucleosomes: 832 ± 70 vs. 52 ± 26 mOD for10^9^ neutrophils/L, *p* < 0.05; citrullinated histone H3/DNA complexes: 242 ± 180 versus 76 ± 30 mOD for10^9^ neutrophils/L, *p* < 0.05; neutrophil elastase/DNA complexes: 227 ± 48 versus 90 ± 27 mOD for10^9^ neutrophils/L, *p* < 0.05). rhTM significantly prevented the increase in circulating NETs evaluated as complexes between either citrullinated antihistone H3 and DNA, or neutrophil elastase and DNA in septic compared to placebo (citrullinated antihistone H3-DNA: 93 ± 16 vs. 242 ± 180 mOD for 10^9^ neutrophils/L, *p* < 0.05; neutrophil elastase/DNA: 93 ± 33 vs. 227 ± 48 mOD for10^9^ neutrophils/L, *p* < 0.05) (Fig. 4).

## Discussion

According to the recently described immunothrombosis concept [8], adequate and controlled coagulation activation would stand for an essential innate immune defense mechanism. When over-activated, coagulation pathways however switch from a beneficial to an uncontrolled noxious response. Similarly, excessive neutrophil activation could have deleterious consequences, notably by sustaining coagulation through NETs formation. In the present study, we report that rhTM both impact coagulation and neutrophil activation, two main actors of immunothrombosis.

Sepsis is indeed always associated with the activation of the coagulation cascade [28], resulting in excessive thrombin formation, defective fibrinolysis and consumption of natural anticoagulant proteins, ultimately leading to DIC. Nevertheless, impairment of fibrinolysis plays a key role in microvascular thrombosis, responsible for multiple organ failure syndrome and death [29]. Delayed fibrinolysis might therefore also contribute to septic shock-induced coagulopathy and DIC. In the present study, we demonstrated that rhTM decreased septic shock-induced coagulopathy and was associated with reduced consumption of both platelets and natural coagulation inhibitors. Interestingly, besides its direct and expected anticoagulant effects, this is the first report showing that rhTM limits the release of MVs and thereby coagulation supported by their procoagulant surface. These observations are consistent with previously reported data from the laboratory demonstrating that recombinant human activated protein C reduces the generation of procoagulant endothelial and platelet MVs in CPL rats [23]. In sepsis, MVs stand as surrogate markers of blood cell activation and of the procoagulant and pro-inflammatory endothelial state [23, 30]. Interestingly, in the present study, we provide the first evidence that rhTM dramatically enhances (fivefold increase) the proportion of MVs bearing fibrinolytic activity in septic rats, while reducing total MVs by only 50%. These data suggest that rhTM triggers the generation of a MV-borne fibrinolytic activity, MVs being switched from deleterious mediators of coagulopathy to endogenous protective effectors. Lacroix et al. [4] previously showed that MVs circulating in patients suffering from vascular diseases bore a fibrinolytic activity. Although we have assessed the fibrinolytic activity borne by total MVs and not by MV subtype, it is interesting to note that the ratio of fibrinolytic activity to leukocyte-derived MVs is significantly increased (data not shown), conversely to the ratio of fibrinolytic activity to endothelial or platelet-derived MVs, and is therefore in favor of a leukocyte-dependent plasmin generation restored by rhTM, most probably through the generation of uPAR highly exposing leukocyte MVs. Yet we cannot exclude that endothelial MVs known to expose tPA, another activator of plasmin generation, could also contribute to a rhTM-restored plasmin activity [4].

MVs have also been identified as early and relevant biomarkers of sepsis-induced DIC in humans [31]. We have indeed recently shown that the leukocyte-derived CD11a^+^-MVs-to-leukocyte count ratio was significantly associated with DIC [31], confirming the role of leukocytes, and mainly of neutrophils, in DIC pathogenesis [7, 32, 33]. In line with these former results, we herein evidence a leukocyte-derived MVs-to-leukocyte count ratio significantly increased in rats with septic shock that is reduced by rhTM treatment. Anticoagulation by rhTM during septic shock would therefore decrease leukocyte activation and consecutive inflammatory processes which are consistent with other data suggesting that anticoagulant treatment may decrease excessive inflammation and/or be cytoprotective [15, 34].

Since we recently brought evidences that NETosis is an early marker of DIC during septic shock [33], our aim was to modulate NETosis by anticoagulation therapy in order to limit exaggerated immunothrombosis. We herein confirm that circulating indirect markers of NETosis were significantly decreased by rhTM treatment in septic rats.

The role of NETosis in coagulopathy and the mechanism by which NETs promote thrombosis, however, remain a matter of debate. Noubouossie et al. [35] shed considerable doubt on the role of NETs in coagulation. In their in vitro study, human NETs isolated from normal individuals indeed failed to directly initiate or amplify coagulation, unlike DNA or isolated histone proteins. Authors suggested that complex interactions between histones and DNA within the nucleosome unit might have been altered by NETs isolation, the latter being, however, not performed from septic samples. Moreover, NETs were reported to promote thrombosis in various animal models [36–38] and we recently showed that circulating NETs are associated with DIC in septic shock patients [32]. Similarly, interaction of NETs with platelets/inorganic polyphosphate was shown to induce intravascular coagulation during sepsis in mice, while blockade of in vivo NETosis decreased coagulopathy and organ injury [39]. In the present study, even though leukocyte activation and NETs contribution to septic shock-induced coagulopathy was evidenced by indirect markers, both were significantly associated with coagulopathy and their levels limited by rhTM treatment. To our knowledge, this is the first report of a protective effect of thrombomodulin in septic shock-induced NETosis. Interestingly, the degree of NETosis was associated with the histological lung score evaluating the severity of lung damages, suggesting a pivotal role of exaggerated NETosis in organ injury. NETosis might therefore take part to septic shock-induced multiple organ failure and, in our hands, is at least partly counteracted by rhTM treatment.

In spite of these encouraging results, inhibiting the immunothrombosis process during sepsis may be deleterious, as it constitutes a first line host defense against pathogens. Yet, we propose here that only excessive and deregulated immunothrombosis should be counteracted. In our septic shock model with coagulopathy, rats thus seem to benefit from immunothrombosis down regulation, while they would probably not in a less severe form of an infection without excessive coagulation activation.

Our study suffers some limitations. First, DIC cannot be defined by a specific score in a rat septic shock model. Even in clinical practice, DIC diagnosis is difficult using the available scores and there is no real gold standard yet [14]. Then, most of the parameters have been evaluated at one time point during the study, as rat volemia would not allow us iterative blood samplings.

While there is growing evidence that neutrophil activation is a keystone in host defense and immunothrombosis, our data demonstrate the feasibility of a pharmacological normalization of exaggerated immunothrombosis most probably by rescuing anticoagulant natural pathways. Altogether, we bring evidence that treating rats with rhTM during septic shock limits excessive neutrophil activation and rescues a balanced coagulation and immunothrombosis response. Ultimately, our study indicates that neutrophils stand as a promising new therapeutic target in the control of coagulopathy during septic shock.

## Additional file

**Additional file 1: Figure S1.** Protocol design.

## Abbreviations

CLP: cecal ligation and puncture
DIC: disseminated intravascular coagulation
HE: hematoxylin–eosin
HMGB1: high mobility group box 1
i.p.: intraperitoneal
MVs: microvesicles
NETs: neutrophils extracellular traps
PhtdSer: phosphatidylserine
rhTM: recombinant human thrombomodulin
uPAR: urokinase-type plasminogen activator receptor
tPA: tissue plasminogen activator
