# Supplementary figures and images for: Thrombomodulin favors leukocyte microvesicle fibrinolytic activity, reduces NETosis and prevents septic shock-induced coagulopathy in rats
Source: Ann Intensive Care. 2017 Dec 8;7:118. doi: 10.1186/s13613-017-0340-z (PMC5722785; doi:10.1186/s13613-017-0340-z)

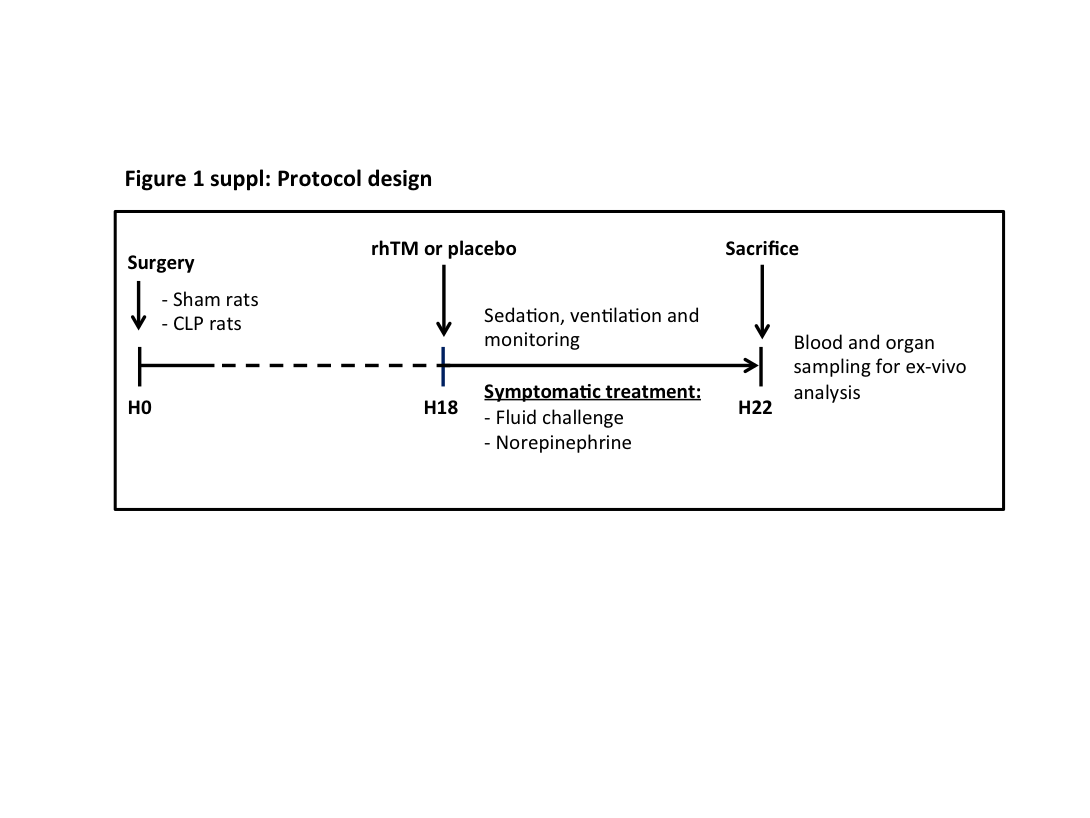

Supplement: Supplementary file 1 — Additional file 1: Figure S1. Protocol design. [file 13613_2017_340_MOESM1_ESM.tif]
